# Sex Determination of Three-Dimensional Skull Based on Improved Backpropagation Neural Network

**DOI:** 10.1155/2019/9163547

**Published:** 2019-01-13

**Authors:** Wen Yang, Xiaoning Liu, Kegang Wang, Jiabei Hu, Guohua Geng, Jun Feng

**Affiliations:** ^1^College of Information Science and Technology, Northwest University, Xi'an, China; ^2^College of Electronics and Information Engineering, Ankang University, Ankang, China

## Abstract

Sex determination from skeletons is a significant step in the analysis of forensic anthropology. Previous skeletal sex assessments were analyzed by anthropologists' subjective vision and sexually dimorphic features. In this paper, we proposed an improved backpropagation neural network (BPNN) to determine gender from skull. It adds the momentum term to improve the convergence speed and avoids falling into local minimum. The regularization operator is used to ensure the stability of the algorithm, and the Adaboost integration algorithm is used to improve the generalization ability of the model. 267 skulls were used in the experiment, of which 153 were females and 114 were males. Six characteristics of the skull measured by computer-aided measurement are used as the network inputs. There are two structures of BPNN for experiment, namely, [6; 6; 2] and [6; 12; 2], of which the [6; 12; 2] model has better average accuracy. While *η* = 0.5 and *α* = 0.9, the classification accuracy is the best. The accuracy rate of the training stage is 97.232%, and the mean squared error (MSE) is 0.01; the accuracy rate of the testing stage is 96.764%, and the MSE is 1.016. Compared with traditional methods, it has stronger learning ability, faster convergence speed, and higher classification accuracy.

## 1. Introduction

Forensic anthropologists throughout the world are faced with a tough battle in keeping up with the changing crime behaviour. A possible improvement in counteracting criminal trends is to maximize the available evidence, which may be gleaned from incomplete and often fragmentary skeletal materials. In this regard, sex determination remains a critical aspect of human identification from skeleton in forensic cases as it reduces the number of possible matches by 50%, whilst jointly serving as baseline data for identification procedures such as facial reconstruction [1]. Therefore, sex identification for unknown skeleton is an important work. According to experience and previous studies [2–4], sex classification using pelvis morphological characteristics has the highest accuracy. However, in most cases, we could only get completely skull rather than skeleton, and as skull is composed of hard tissue, it is easily preserved. Therefore, sex identification through the skull has become a core content of forensic anthropology.

The common sex classification includes morphology discriminant method and measurement discriminant method. Traditional sex identification of the skull mainly depends on anthropologists' visual morphology assessment of two state characteristics of sexes and draws conclusions through naked eye observation and experience. Krogman [5] used the morphological method to identify 750 known sex skulls, and the correct rate was 82–87%. Ramsthaler et al. [6] used the kappa statistic to quantify the disagreement between two different observers on gender visual morphology assessment, with a consistency of 90.8% only. With the rapid development of computer technology, computer-aided measurement is increasingly used for the extraction of skull feature items. Shui et al. [7] selected 133 three-dimensional skull models in Xi'an area, measured 14 indexes of skull by computer software, and established multiple sex discriminant functions with the stepwise Fisher method and carried out the back generation test. The male discrimination rate was 87.5%, and the female discrimination rate was 86.7%. Liu [8] analyzed the feature point data of 142 cases of Han skull orthotopic X-ray. Using the SPSS software to analyze, the discriminant regression equation was established and the accuracy rate was 95%. Franklin et al. [9] used OsiriX software to calibrate 31 skulls of 400 skull reconstructions from Australian CT scans, measured 18 characteristics by MorphDb measurement software, and established a gender discriminant function with a recognition accuracy of 90%. Tanya et al. [10] used Sidexis XG software to measure the maxillary sinus of skull radiographs on 50 adult digital skull radiographs. The maxillary sinus index was calculated, and discriminant function analysis was performed. The discriminant equation was determined with a gender of 68%. In summary, we can see that the morphological discrimination method is simple and easy to implement, but it depends too much on expert knowledge and subjective experience, with insufficient theoretical knowledge and low recognition rate. The method of measurement and discrimination is objective, and the recognition rate has been improved, but most of the methods used are based on discriminant analysis to design prediction rules. However, all the results obtained by using these prediction models indicate that the relationship between the probability of an individual belongs to a certain sex and the explanatory variables (bone measurements) are not linear [11, 12].

To solve these problems, in this paper, we propose a method of sex identification based on improved BP neural network. It takes the skull features measured by computer software as input and the result of sex classification as output. By learning the sample, the approximate function relationship between input and output is determined so as to realize gender classification. This is a nonlinear classification method. Unlike DFA, BPNN does not require distributional assumptions of the variables and is able to model all types of nonlinear functions between input and output of a model [13]. The advantages of this method are as follows: firstly, it needs no professional qualification; secondly, it can fully approximate the complex nonlinear relationship of skull data; and finally, it can get a high recognition rate.

## 2. Research Methodology

### 2.1. Materials

This research is carried out on a database of 267 whole-skull CT scans (153 females and 114 males) on voluntary persons that mostly come from the Uighur ethnic group in the north of China (females aged 18–88 and males aged 20–84 ). The images of each subject are restored in DICOM format with a size of approximately 512 × 512 × 250. Each 3D skull surface is extracted from the CT images and is represented as a triangle mesh of about 220,000 vertices. All the skulls are substantially complete; that is, each skull contains all the bones from calvarias to jaw and has full mouth of teeth.

**Figure 1:**
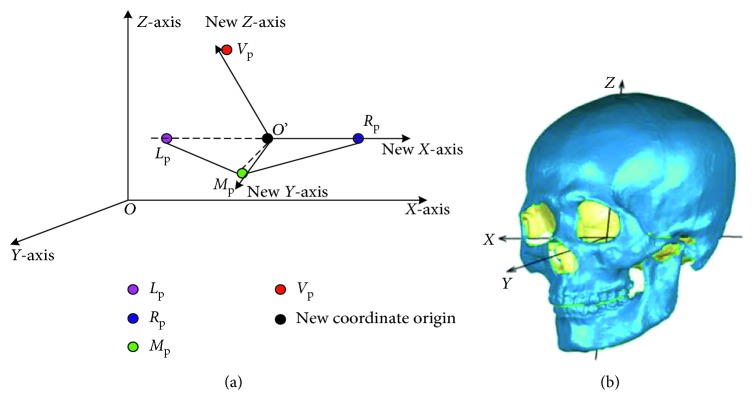
One skull in the uniform coordinate system. (a) Frankfurt coordinate system and (b) skull Frankfurt coordinate system.

All the samples are transformed into a uniform coordinate system so as to eliminate the inconsistence in position, pose, and scale caused by data acquirement. The uniform coordinate system is determined by four skull landmarks, left porion, right porion, left (or right) orbitale, and glabella (denoted as *L*_p_, *R*_p_, *L*_o_, and *G*). The Frankfurt plane [14] is determined by three points, *L*_p_, *R*_p_, and *L*_o_. The coordinate origin (denoted as *O*) is the intersection point of the line *L*_p_ *R*_p_ and the plane that contains point *G* and orthogonally intersects with line *L*_p_ *R*_p_. We take the line *OR*_p_ as *x*-axis. The *z*-axis is the line through the point *O* and with the direction being the normal of the Frankfurt plane. Then, *y*-axis is obtained by the cross product of *z*− and *x* − axis. Once the uniform coordinate system is defined, all the prototypic skulls are transformed into it. Finally, the scale of all the samples is standardized by setting the distance between *L*_p_ and *R*_p_ to unit, i.e., each vertex (*x*, *y*, *z*) of the skull is scaled by (*x*/|*L*_p_ − *R*_p_|, *y*/|*L*_p_ − *R*_p_|, *z*/|*L*_p_ − *R*_p_|). One skull in the uniform coordinate system is shown in Figure 1.

The data used in this paper included 267 skulls consisting of 153 females and 114 males derived from the Visualization Technology Institute of Northwest University in China. The collected data were then measured by computer-aided measurement. There are six variables for gender determination in 3D skull. They are cranial sagittal arc, cranial sagittal chord, apical sagittal arc, apical sagittal chord, occipital sagittal arc, and Occipital sagittal chord. All measurements are represented by symbols, as shown in Table 1.

### 2.2. Backpropagation Neural Network

In this paper, the technical specific BPNN of artificial neural network is proposed for gender determination. ANN can be classified into feed forward and recurrent, according to their connectivity. The ability of ANN to predict outcomes accurately depends on the selection of proper weights in the training. Training or learning is the relationship between inputs and target. The learning rules defined as network processes aim to adjust weights and biases [15]. It uses the rapidest descent to continuously adjust the weights and thresholds of neural network by backpropagation, so as to minimize the sum of the square error of the network [16]. Three types of learning of neural network methods are supervised, unsupervised, and reinforced [17]. In supervised learning, the network is provided with inputs and desired outputs or target values. In unsupervised learning, on the other hand, the weights and biases are modified only through response to network inputs, using mean squared error (MSE) to measure the performance of the models. MSE is the average of the squares of the difference between each output and the desired output, given by the following equation:

(1) $$\begin{array}{c} \text{MSE}=\frac{1}{n}\overset{n}{\underset{i=1}{\sum }}\overset{yd}{\underset{j=1}{\sum }}{\left(y_{j}^{i}-{\hat{y}}_{j}^{i}\right)}^{2}. \end{array}$$

**Figure 2:**
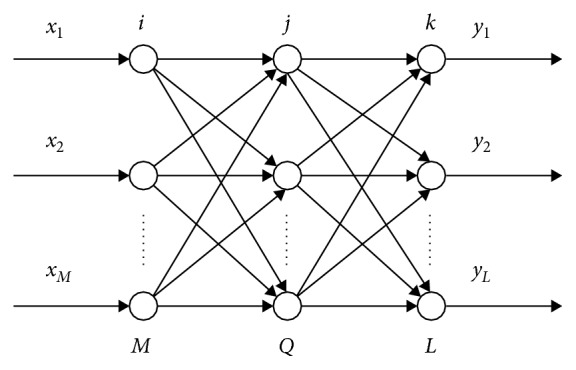
BP neural network model.

ANN is learned by the backpropagation algorithm in which the errors of the hidden layer units are determined by the errors of the output layer units [18]. The self-learning of BP neural network usually has two parts: one is the forward transmission of information; another is the reverse transmission of error between excepted output and actual output. The structure of BP neural network consists of three parts: input layer, hidden layer, and output layer. The model of BP neural network is shown in Figure 2 [19].

There are *M* neurons in the input layer of the network, *Q* neurons in the hidden layer, and *L* neurons in the output layer. The input vector of neural network is *X*=[*x*_1_, *x*_2_, ..., *x*_*M*_], and the output vector is *Y*=[*y*_1_, *y*_2_, ..., *y*_*L*_]. The weighted value between input layer and hidden layer is *w*_*ij*_, and the weighted value between hidden layer and output layer is *w*_*jk*_. The transfer function of neural network is unipolar sigmoid function which is *f*(*x*)=1/1+*e* ^−*x*^. The function has the characteristic which is *f*′(*x*)=*f*(*x*)[1 − *f*(*x*)] [20].

In accordance with the gradient descent method, the data transmit from the input layer to the hidden layer which is *O*_*i*_=*x*_*i*_. After the hidden layer receives the data from the input layer, the first thing we should do is weighted sum as net_*j*_=∑_*i*=1_^*M*^ *w*_*ij*_ *x*_*i*_. And then, the data are transferred to the output layer through the transfer function. The output of hidden layer is *O*=*f*(net_*j*_)=*f*(∑_*i*=1_^*M*^ *w*_*ij*_ *x*_*i*_).

The learning rules for the standard BP network are self-learning weighted coefficients including the weighted coefficient between the input layer and hidden layer and the weighted coefficient between the hidden layer and output layer. The following are the two rules:

(2) $$\begin{array}{c} \Delta w_{ij}=-\eta \frac{\partial E}{\partial w_{ij}},\\ \Delta w_{jk}=-\eta \frac{\partial E}{\partial w_{jk}},\\ w_{jk}\left(t+1\right)=w_{jk}\left(t\right)+\Delta w_{jk}, \end{array}$$

where *η* is the learning rate and *d*_*k*_ is the expectation output value. *E*=(1/2)∑_*k*=1_^*L*^(*d*_*k*_ − *y*_*k*_)^2^ is the error [21].

The following is the calculation method of BP neural network to adjust the weighted coefficients Δ*w*_*jk*_ and Δ*w*_*ij*_:

(3) $$\begin{array}{c} \Delta w_{jk}=-\eta \frac{\partial E}{\partial w_{jk}}=-\eta \frac{\partial E}{\partial w_{jk}}\frac{\partial O_{k}}{\partial {\text{net}}_{k}}\frac{\partial {\text{net}}_{k}}{\partial w_{jk}},\\ \Delta w_{jk}=\eta \left(d_{k}-O_{k}\right)O_{k}\left(1-O_{k}\right)O_{j},\\ \Delta w_{ij}=-\eta \frac{\partial E}{\partial O_{k}}\frac{\partial O_{k}}{\partial {\text{net}}_{k}}\frac{\partial {\text{net}}_{k}}{\partial O_{j}}\frac{\partial O_{j}}{\partial {\text{net}}_{j}}\frac{\partial {\text{net}}_{j}}{\partial w_{ij}},\\ \Delta w_{ij}=\eta O_{j}\left(1-O_{j}\right)O_{i}\overset{L}{\underset{k=1}{\sum }}\left[\left(d_{k}-O_{k}\right)O_{k}\left(1-O_{k}\right)w_{jk}\right]. \end{array}$$

### 2.3. Improved Backpropagation Neural Network

In practical applications, there are many shortcomings in the basic BP neural network algorithm. The commonly recognized problem is that the convergence speed is slow and it is easy to fall into a local minimum. In addition, there are still shortcomings of poor stability and low generalization ability. This method improves the deficiencies of the BP neural network algorithm.

#### 2.3.1. Introduction of Momentum Term

The momentum term is added to improve the convergence rate and avoid falling into local minimum. The selection of learning step in the BP algorithm is very important. The convergence speed of the network increases with the increase of *η* value, but if the *η* value is too large, it will cause oscillation instability. The easiest way to solve this problem is to add a momentum term, that is,

(4) $$\begin{array}{c} \Delta \omega_{ij}\left(n+1\right)=\alpha \Delta \omega_{ij}\left(n\right)+\eta \delta_{j}\left(n\right){ο}_{j}, \end{array}$$

where *α* is a momentum term, usually an integer, and (*n*+1) represents the (*n*+1)th iteration. *ηδ*_*j*_(*n*)*ο*_*j*_ indicates that the (*n*+1)th correction of *ω*_*ij*_ should keep the *n*th correction to a certain extent. Adding momentum in the BP algorithm can not only fine tune the correction amount of connection weights and accelerate the convergence speed but also avoid falling into local minima [22–24].

#### 2.3.2. *L*_2_ Regularization Method

The BP neural network has the characteristics of weak stability, which makes the gender prediction value misjudged in the case of little difference in skull characteristics. Considering the overlap of skull sex determination, this paper proposes adding the *L*_2_ regularization term to the objective function to make the model more stable. After adding regularization, the objective function of the BP neural network becomes

(5) $$\begin{array}{c} \min\text{:}\;\;\frac{1}{2}{\left\|y_{p}-y_{r}\right\|}_{2}^{2}+\lambda {\left\|C\right\|}_{2}^{2}, \end{array}$$

where *y*_p_ is the gender classification predicted by the model and *y*_r_ is the true sex classification. *λ* is the regularization coefficient. ‖*C*‖_2_^2^ is a regularization term, and its calculation method is to calculate the square of the ownership value and then find the square root.

#### 2.3.3. Adaboost Integration Algorithm

In addition to the above possible problems, the BP neural network is still too sensitive and the model generalization ability is not strong enough. Adaboost is a relatively mature and widely used ensemble algorithm, which can significantly improve the accuracy and generalization ability of the algorithm [25]. Several BP neural networks are combined to make the neural networks complementary. The final result of the algorithm is weighted by the results of all BP neural networks. For *N* training samples ((*x*_1_, *y*_1_, *z*_1_), (*x*_2_, *y*_2_, *z*_2_),…, (*x*_*N*_, *y*_*N*_, *z*_*N*_)), *T* (specifically artificially given) BP neural networks are established. Then, the initial weight of the sample is set as follows:

(6) $$\begin{array}{c} D_{t}\left(i\right)=\frac{1}{N}, \end{array}$$

where *D*_*t*_(*i*) represents the weight of the sample in the *t* iteration.

Under the *D*_*t*_(*i*), the weak learner *h*_*t*_(*x*) is trained (that is, the *t*th BP neural network), and the error *ε*_*i*_ and average error *ε*_*t*_ of each sample are calculated. *ε*_*i*_ and *ε*_*t*_ are used to calculate the weight of the current weak learner and update the sample weight of the next iteration (that is, the (*t*+1)th BP neural network):

(7) $$\begin{array}{c} \left\{\begin{array}{c} W_{t}=\frac{1}{2}\ln\left(\frac{\varepsilon_{t}}{1-\varepsilon_{t}}\right),\\ D_{t+1}\left(i\right)=\frac{D_{t}\left(i\right){\left(\varepsilon_{t}/1-\varepsilon_{t}\right)}^{-\varepsilon_{i}}}{\sum_{i=1}^{n}\left[D_{t}\left(i\right){\left(\varepsilon_{t}/1-\varepsilon_{t}\right)}^{-\varepsilon_{i}}\right]}, \end{array}\right. \end{array}$$

where *W*_*t*_ is the weight of the *t*th weak learner. *D*_*t*+1_(*i*) is the weight of the *t*+1 BP neural network samples.

The above steps are iterated *T* times to obtain the Adaboost integrated prediction method. When forecasting, each weak learner is weighted to get the final prediction result:

(8) $$\begin{array}{c} H\left(x\right)=\overset{T}{\underset{t=1}{\sum }}W_{t}h_{t}\left(x\right). \end{array}$$

Using the improved BPNN algorithm, we can get more accurate results than other single nonlinear models.

## 3. Discussion

The data used are 267 skulls, including 153 females and 114 males. The data collection is measured using the metric method. The data are measured and stored in the Excel table. After measurement, the BPNN was developed in MATLAB R2012a. The data were normalized and then divided into 70% for training and 30% for testing. In this step, we just need to explain the BPNN technology, without describing the DFA technique, because it is only used as a comparison of the final results.

**Figure 3:**
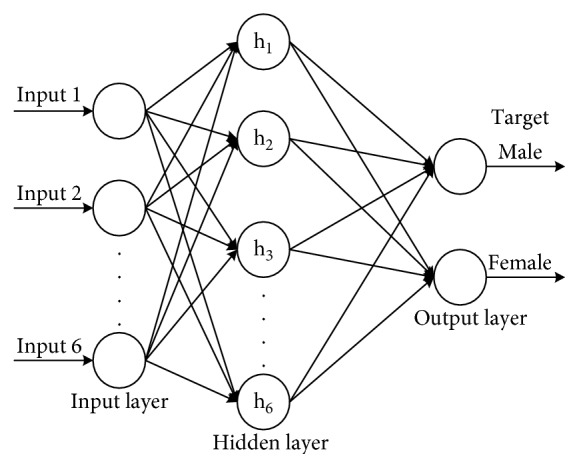
The architecture of BPNN with the hidden layer consisting of 6 neurons.

**Figure 4:**
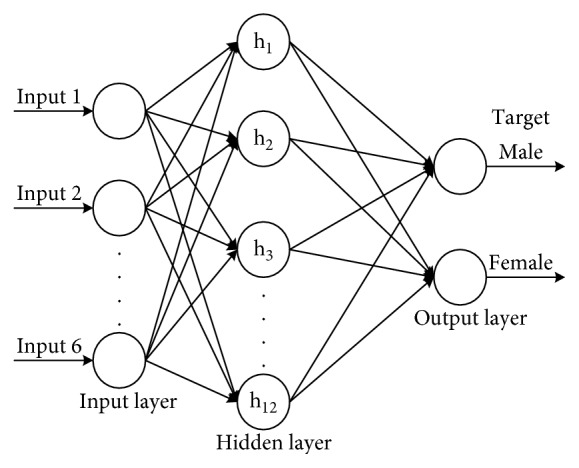
The architecture of BPNN with the hidden layer consisting of 12 neurons.

As a first step, the architecture of the network has to be decided. The architecture of BPNN for case is divided into two models, namely, [6; 6; 2] and [6; 12; 2]. The architectures in this research are shown in Figures 3 and 4. In addition, we build 4 BP neural networks for training samples and iterate 4 times to get the Adaboost-integrated BP neural network model. Due to the characteristics of the Adaboost algorithm, the samples have different weights for different neural networks. By simulation, the weight of the Adaboost algorithm is [0.312, 0.426, 0.534, 0.713] in the neural network structure [6; 6; 2] and [0.423, 0.566, 0.696, 0.754] in the neural network structure [6; 12; 2]. The regularization coefficient is set to 2^25^.

Figures 3 and 4 demonstrate that the BPNN structure used in this example is made up of six inputs based on skull variables (CSA, CSC, ASA, ASC, OSA, and OSC). The hidden layer given in Figure 3 consists of 6 neurons, and the hidden layer given in Figure 4 consists of 12 neurons. The output layer consisted of two neurons, namely, female and male. After designing the layering of BPNN, the calculating process of BPNN is developed in MATLAB R2012a.

Before learning process, parameters to be used must be defined. In this research, learning process was stopped after 100,000 iteration epochs using log-sigmoid for activation function, and momentum (*α*) was 0.1; 0.5; 0.9 and learning rate (*η*) was 0.1; 0.5; 0.9 (Table 2). Computing error in the output layer was backpropagated to earlier ones in order to update the current input-hidden layer weights and output-hidden layer weights. By updating these weights, the network would learn to reach the target. The target reached is 1 for female and 0 for male. In the algorithm, the error was calculated in the output, and the new values of weights were computed in each layer until the error was minimized to a considerable value. The measurement of ANN performance was observed by using the MSE and total prediction accuracy of the network to the tested data. And, training is best when the ANN is capable to achieve the lowest MSE value.

In the learning process of BPNN, the experiment repeats 10 times and the results are outlined in Tables 3 and 4.

Table 3 describes the best training and testing results obtained by performing the experiment of the structural model 10 times [6; 6; 2]: *η* = 0.9 and *α* = 0.9. The average accuracy obtained for training is 96.145% and testing is 95.336%.

The results of the structural model [6; 12; 2] can be seen in Table 4. It indicates that the performance of each *η* and *α* yields different results in both training and testing. The experiment was repeated 10 times. The highest accuracy was found while *η* = 0.5 and *α* = 0.9, namely, 97.232% and 96.764% of the training and testing classification rates, respectively.

**Figure 5:**
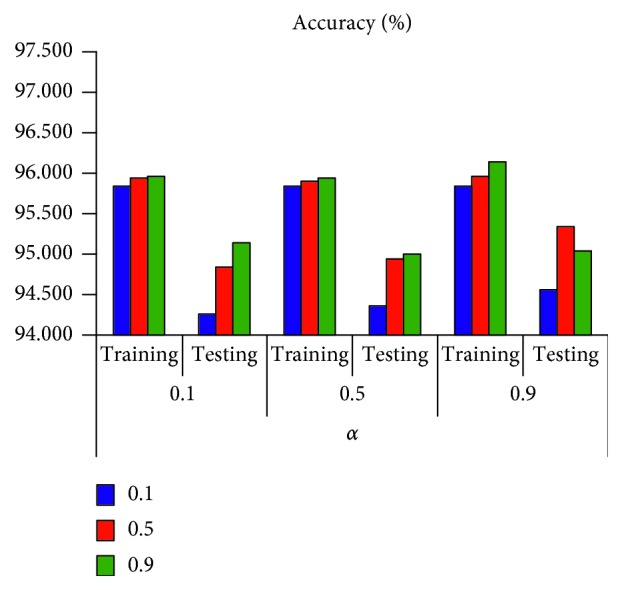
The performance of structure [6; 6; 2].

**Figure 6:**
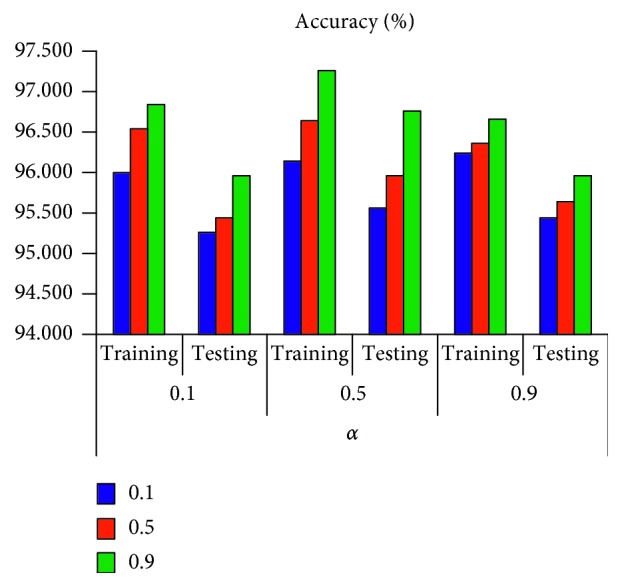
The performance of structure [6; 12; 2].

The average accuracy results of the two structural models are shown in Figures 5 and 6. The results of the structural model [6; 6; 2] can be seen in Figure 5. The average accuracy of the training phase is higher than the test phase. The results of the structural model [6; 12; 2] can be seen in Figure 6. The average accuracy of the training phase is also higher than the testing phase. Comparing the results of the two structural models, we can see that, for the same *η* and *α*, the average accuracy of the training phase and the testing phase of the structural model [6; 12; 2] is higher than that of the structural model [6; 6; 2].

The comparison between the BP neural network and standard classification techniques for sexual dimorphism, that is, univariate and multivariate discriminant analysis (using six variables) and logistic regression (using six variables), are presented in Table 5. The BP neural network using the six variables had an accuracy rate of 96.764%.

In this paper, two classic sex determination methods (i.e., discriminant analysis and logistic regression) were compared with an artificial neural network. The BP neural network using all six variables gives the best overall results (96.764%) and achieves the highest rate of correctly classified individuals. Mahfouz et al. [11] used the linear discriminant classification method for patella to get a correct classification rate of 90.3%, while using feed forward backpropagation neural network to get 96% classification accuracy. Usually, the correct rate of sex classification for patellae is only about 85% [26, 27]. These results reflect other studies that neural networks with better results than other linear methods (e.g., logistic regression and discriminant analysis).

## 4. Conclusion

This paper presents a complete classification framework for gender determination in forensic anthropology. After analyzing the standard BP neural network algorithm, we propose an improved BP neural network algorithm, which points out the disadvantages with the algorithms above. It adds the momentum term to improve the convergence speed and avoids falling into local minimum. The regularization operator is used to ensure the stability of the algorithm, and the Adaboost integration algorithm is used to improve the generalization ability of the model. The final experiment shows that the [6; 12; 2] structure of BPNN achieves the best results in the skull data set of this paper, namely, 97.232% training and 96.764% testing. Compared with other classification techniques, BPNN can improve the result of gender determination with providing high-accuracy result. Moreover, although we use CT scans to construct 3D-point cloud model of the skull in this work, the BPNN model we build can also deal with 3D models constructed in any way such as laser scan 3D camera. Next, we should collect a larger sample to build a neural network-based model that will be implemented for practical applications of sex assessment of an unknown bone in forensic cases.

In the future work, in terms of gender determination, classification techniques can be combined to provide higher accuracy and better techniques.

## Figures and Tables

**Table 1:** The variables for measurement of skulls.

Description of variables	Variables
Cranial sagittal arc	CSA
Cranial sagittal chord	CSC
Apical sagittal arc	ASA
Apical sagittal chord	ASC
Occipital sagittal arc	OSA
Occipital sagittal chord	OSC

**Table 2:** Parameters learning rate (*η*) toward momentum (*α*) in BPNN.

No.	*lr*	*α*
1	0.1	0.1
2		0.5
3		0.9

4	0.5	0.1
5		0.5
6		0.9

7	0.9	0.1
8		0.5
9		0.9

**Table 3:** Experimental result of BPNN for training and testing [6; 6; 2].

	*η*	*α*		
		0.1	0.5	0.9
*Performance training*				
Accuracy (%)	0.1	95.837	95.916	95.987
MSE		0.014	0.013	0.010

Accuracy (%)	0.5	95.851	95.921	95.994
MSE		0.013	0.013	0.011

Accuracy (%)	0.9	95.887	95.946	**96.145**
MSE		0.013	0.012	0.010

*Performance testing*				
Accuracy (%)	0.1	94.269	94.812	95.146
MSE		0.022	0.018	0.021

Accuracy (%)	0.5	94.377	94.921	94.994
MSE		0.029	0.023	0.018

Accuracy (%)	0.9	94.566	**95.336**	95.028
MSE		0.017	0.014	0.019

**Table 4:** Experimental result of BPNN for training and testing [6; 12; 2].

	*η*	*α*		
		0.1	0.5	0.9
*Performance training*				
Accuracy (%)	0.1	96.006	96.531	96.824
MSE		0.012	0.010	0.011

Accuracy (%)	0.5	96.134	96.618	**97.232**
MSE		0.012	0.011	0.010

Accuracy (%)	0.9	96.261	96.357	96.669
MSE		0.014	0.010	0.010

*Performance testing*				
Accuracy (%)	0.1	95.298	95.472	95.994
MSE		0.998	1.236	1.252

Accuracy (%)	0.5	95.563	95.981	**96.764**
MSE		0.954	1.387	1.016

Accuracy (%)	0.9	95.476	95.617	95.973
MSE		1.604	1.229	1.547

**Table 5:** Comparison between the BPNN and other classification models.

	Classification method	% of skull accuracy classified		
		*M*	*F*	Mean
Multivariate	BPNN (6 variables: CSA, CSC, ASA, ASC, OSA, and OSC)	96.764	96.764	96.764

Univariate	Discriminant analysis (CSA)	78.5	78.1	78.3
	Discriminant analysis (CSC)	77.8	76.5	77.2
	Discriminant analysis (ASA)	75.3	73.9	74.6
	Discriminant analysis (ASC)	75.1	71.6	73.4
	Discriminant analysis (OSA)	74.9	76.1	75.5
	Discriminant analysis (OSC)	74.7	75.4	75.1

Multivariate	Discriminant analysis (6 variables: CSA, CSC, ASA, ASC, OSA, and OSC)	87.8	88.4	88.1
	Logistic regression (6 variables: CSA, CSC, ASA, ASC, OSA, and OSC)	89.6	91.2	90.4

## Data Availability

The .obj format 3D model data used to support the findings of this study may be released upon application to the Northwest University Visual Technology Institute via the email xnliu@nwu.edu.cn.

## References

[1] Loth S. R., Iscan M. Y., Knupfer G. C. (2000). Sex determination. *Encyclopedia of Forensic Sciences*.

[2] Snow C. C. (1982). Forensic anthropology. *Annual Review of Anthropology*.

[3] Spradley M. K., Jantz R. L. (2015). Sex estimation in forensic anthropology: skull versus postcranial elements. *Journal of Forensic Sciences*.

[4] Williams B. A., Rogers T. L. (2006). Evaluating the accuracy and precision of cranial morphological traits for sex determination. *Journal of Forensic Sciences*.

[5] Krogman W. M. (1962). *Book Reviews: The Human Skeleton in Forensic Medicine*.

[6] Ramsthaler F., Kettner M., Gehl A., Verhoff M. A. (2010). Digital forensic osteology: morphological sexing of skeletal remains using volume-rendered cranial CT scans. *Forensic Science International*.

[7] Shui W. Y., Yin R. C., Zhou M. Q. (2013). Sex determination from digital skull model for the Han people in China. *Chinese Journal of Forensic Medicine*.

[8] Liu Y. Y. (2016). The sex determination of Han nationality in North China by adult facial skull X-ray. *Chinese Journal of Forensic Sciences*.

[9] Franklin D., Cardini A., Flavel A., Kuliukas A. (2013). Estimation of sex form cranial measurements in a Western Australian population. *Forensic Science International*.

[10] Tanya K., Arpita K., Uday G., Jain R. (2017). Cephalometric analysis for gender determination using maxillary sinus index: a novel dimension in personal identification. *International Journal of Dentistry*.

[11] Mahfouz M., Badawi A., Merkl B. (2007). Patella sex determination by 3D statistical shape models and nonlinear classifiers. *Forensic Science International*.

[12] Walker P. L. (2008). Sexing skulls using discriminant function analysis of visually assessed traits. *American Journal of Physical Anthropology*.

[13] Du J. P., Ponsaille J., Alunni-Perret V., Quatrehomme G. (2009). A comparison between neural network and other metric methods to determine sex from the upper femur in a modern French population. *Forensic Science International*.

[14] The Wikimedia Foundation. Inc. Web http://en.wikipedia.org/wiki/Frankfurt_plane.

[15] Demuth H., Beale M. (2009). Neural network toolbox. *Mathworks*.

[16] Zhou J. G., Qin G. Application of BP neural network forecast model based on principal component analysis in railways freight forecas.

[17] Kumaravel G., Kumar C. A novel bats echolocation system based back propagation algorithm for feed forward neural network.

[18] Kottaimalai R., Rajasekaran M. P., Selvam V., Kannapiran B. EEG signal classification using principal component analysis with neural network in brain computer interface applications.

[19] Rumerlhar D. E., Hinton G. E., Williams R. J. (1986). Learning representation by back-propagating errors. *Nature*.

[20] Fletcher R., Reeves C. M. (1964). Function minimization by conjugate gradients. *Computer Journal*.

[21] Smagt P. (1994). Minimisation methods for training feedforward neural networks. *Neural Networks*.

[22] Yang X. M., Liu R. L. (2006). Identification of carbonate reservoir by neural network model. *Inner Mongolia Journal of petroleum and chemical industry in China*.

[23] Luo L., Yao S. X., Ren X. G. (2002). Applications of numerical calculation and model identification technology based on neural network in log interpretation. *WLT*.

[24] Hou J. S., Wei Z. L. (1996). Application of self-organizing neural network to logging data interpretation. *WLT*.

[25] Li Z. C., Sun J. M., Geng S. C. (2001). Classification of fractured reservoirs by spectrum of nuclear magnetic resonance log. *Geophysical Prospecting for Petroleum*.

[26] Introna F., Vella G. D., Campobasso C. P. (1998). Sex determination by discriminant analysis of patella measurements. *Forensic Science International*.

[27] Dayal M. R., Bidmos M. A. (2005). Discriminating sex in South African blacks using patella dimensions. *Journal of Forensic Sciences*.

